# Coating Polyetheretherketone Implant Surface With Titanium Oxide Nanoparticles and Hyaluronic Acid: An In Vitro Study

**DOI:** 10.7759/cureus.77383

**Published:** 2025-01-13

**Authors:** Mohammed Aso Abdulghfor, Mohammed Khalid Mahmood, Zana Fuad Noori, Handren Ameer Kurda, Balen Hamid Qadir, Kawan Salah Othman, Mohammed Taib Fatih, Soma Amin, Nwsiba Khalid Mahmood

**Affiliations:** 1 Oral and Maxillofacial Surgery, Sulaimani University, Sulaimani, IRQ; 2 Odontology, Aix-Marseille University, Marseille, FRA; 3 Oral Biology, American University of Iraq-Sulaimani, Sulaimani, IRQ; 4 Orthodontics, Sulaimani University, Sulaimani, IRQ; 5 Prosthodontics, Komar University of Science and Technology, Sulaimani, IRQ; 6 Prosthodontics, Sulaimani University, Sulaimani, IRQ; 7 Periodontics, Komar University of Science and Technology, Sulaimani, IRQ; 8 Histopathology, Faruk Medical City, Sulaimani, IRQ; 9 Department of Biology, University of Sulaimani, College of Science, Sulaymaniyah, IRQ

**Keywords:** dental implant, hyaluronic acid, mg-63 cell line, osseointegration, peek, polyetheretherketone, surface coating, titanium oxide

## Abstract

Background

Recently, polyetheretherketone (PEEK) has emerged as a promising implant material with some limitations. The objective of this investigation was to improve the hydrophilicity and biocompatibility of PEEK implant material through surface coating by nanoporous titanium oxide (TiO_2_) and hyaluronic acid (HA), targeting a higher degree of cell viability, proliferation, differentiation, and mineralization around PEEK implants.

Methods

Prior to titanium film deposition, the PEEK substrates were treated with argon plasma. Using plasma sputtering, the 800 nm thick TiO_2_ coatings were applied to the plasma-treated PEEK. The PEEK surface was then coated with a layer of HA. The implant discs were seeded with osteoblast cells. A total of four groups, each containing eight discs, were created. The groups included uncoated PEEK, HA-coated PEEK, HA and nanoporous TiO_2 _coated PEEK, and the control group, which contained osteoblast cells only. The 3-[4,5-dimethylthiazol-2-yl]-2,5 diphenyl tetrazolium bromide (MTT) test, 4',6-diamidino-2-phenylindole (DAPI) staining method, alkaline phosphatase (ALP) activity, and alizarin red staining were used to assess cell viability, proliferation, differentiation, and mineralization capacity.

Results

Osteoblast proliferation rates of the HA nanoporous TiO_2_ coated group (69.37±6.16), were greater than HA-coated group (60.12±2.41), the uncoated PEEK (53.25±2.37) and the control group (36.37±4.56) after 21 days according to DAPI test. Additionally, the ALP activity on HA nanoporous TiO_2_ coated PEEK (0.59±0.15, 2.18±0.23) was higher than HA-coated PEEK disks (0.62±0.17, 1.43±0.16), the uncoated group (0.45±0.11, 0.84±0.24) and the control group (0.17±0.08, 0.32±0.16) after seven and 21 days, respectively. Alizarin red staining in the HA nanoporous TiO_2_ coated PEEK (1.41±0.07, 1.72±0.11) was significantly higher than HA-coated PEEK group (1.24±0.11, 1.35±0.07), the uncoated group (1.20±0.10, 1.01±0.08) and the control group (1.00±0.09, 1.00±0.05) at both time intervals, respectively. However, HA-coated PEEK increased cell viability (1.14±0.07, 1.14±0.07) more than the HA nanoporous TiO_2_ coated group (1.10±0.40, 1.10±0.40), uncoated PEEK group (0.99±0.07, 0.99±0.07) and the control group (1.00±0.01, 1.00±0.01) after 72 hours and seven days, respectively.

Conclusion

Double coating of HA and nanoporous TiO_2_ on the PEEK implant surface increased its biofunctional properties through the proliferation, differentiation, and mineralization of osteoblasts.

## Introduction

An implant has become a standard protocol for the replacement or support of function and esthetic of a biological structure, and it is gaining popularity owing to its high success rate and predictable results. The effectiveness of implants depends on creating a direct and stable interaction between the implant surface and the surrounding bone tissue, known as osseointegration [1]. A variety of factors, including the material and surface qualities of the implant, as well as the biological reaction of the host tissue, are important in determining the outcome of the osseointegration process [2].

Polyetheretherketone (PEEK) has emerged as a possible alternative to standard titanium alloys used in bone implants. Its mechanical qualities are similar to those of bone, minimizing the problems associated with stiffer materials like titanium. Furthermore, PEEK has low friction, corrosion resistance, and radiolucency, making it an appealing alternative for a variety of therapeutic applications [3,4].

However, the osseointegration capabilities and long-term clinical success of PEEK as an implant material need to be improved. The hydrophobic nature and low surface energy of PEEK have been identified as the factors that restrict the adhesion and proliferation of bone cells on the implant surface, as various studies report its osseointegration limitations [5,6].

The application of appropriate bioactive coatings, such as biocompatible coatings, osteoconductive coatings, hard coatings, coatings for sustainable antibiotic release, antimicrobial coatings, antimicrobial paints in clinical settings, and corrosion-resistant coatings, are promising methods for attaining optimal surface qualities in implant materials. These coatings improve the implants' mechanical and biological qualities [2,7]. In the past two decades, the utility of titanium film as a novel bioactive coating that can be directly deposited onto metallic (cobalt chromium alloy) and polymeric implants without causing surface injury has been demonstrated. Furthermore, the biocompatibility of implants could be significantly improved by anodizing the titanium coatings that are deposited onto them, resulting in the formation of highly nanoporous titanium oxide (TiO_2_) surfaces [8,9].

Hyaluronic acid (HA), which is already produced by the human body, is one of the substances that has lately been considered for use on the surface of implants. This glycosaminoglycan, which is a part of the extracellular matrix, makes sense due to its secondary stability, osteoconductivity, and positive interactions with the bone progenitor cells [10,11].

The objective of this investigation was to improve the hydrophilicity and biocompatibility of PEEK implant material through surface coating by nanoporous TiO_2_ and HA, targeting a higher degree of cell viability, proliferation, differentiation, and mineralization around PEEK implants.

## Materials and methods

Ethical consideration

This research was approved by the ethics committee of Sulaimani University (no: 119/2023).

Sample preparation

PEEK disks (10 mm in diameter and 1 mm in thickness) were used as the specimens. High-resolution helical CT scanning (Philips, Amsterdam, Netherlands) was used to obtain the hand data, which was then imported into Materialise Mimics 21.0 (Materialise, Leuven, Belgium) to create point cloud data. The data was then saved in STL format. Ultimately, the PEEK disk data was fed into the fused deposition modeling (FDM) system after being imported into a cura slicer (UltraMaker Cura, USA) for slicing. The disks were then produced using an Essentium HSE 240T 3D printer (Essentium, Pflugerville, USA). The printing parameters, which included layer thickness, nozzle diameter, nozzle temperature, and printing speed, were set at 0.2 mm, 0.4 mm, 420°C, and 10 mm/s, respectively.

Surface Preparation and Protocol for PEEK with Titanium

**Figure 1:**
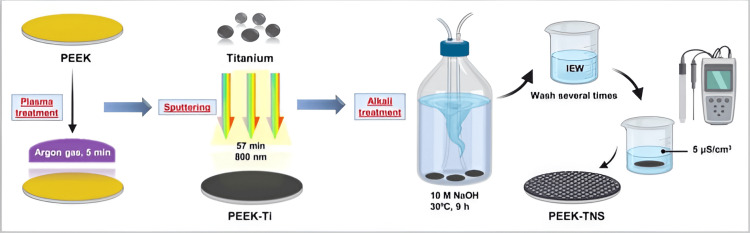
Schematic illustration of the PEEK-Ti and PEEK-TNS sample processing procedures PEEK - polyetheretherketone; Ti - titanium; IEW - ion-exchanged water; NaOH - sodium hydroxide; TNS - titanate nanonetwork structures Source: [12]

For the PEEK control, PEEK disks were ultrasonically cleaned using acetone, ethanol, and deionized water for ten minutes each, and they were gradually ground with sandpaper up to 1500 grit. Prior to titanium film deposition, the PEEK substrates were treated with argon plasma at 30 W and 60 Pa for five minutes. Plasma sputtering (OIKE&Co., Ltd., Kyoto, Japan) was used to deposit the 800 nm thick titanium films on the plasma-treated PEEK for 57 minutes at 250 W, and 9.5×10−2 Pa. Nanoporous TiO_2_ PEEK is a designation for PEEK substrates coated with titanium. The samples were submerged in 10-M sodium hydroxide ​(​​​NaOH) at 30 °C for nine hours in order to create porous nanonetwork structures on their surface. They were then repeatedly washed with ion-exchanged water until the solution achieved a conductivity level [12]. The processing procedure is illustrated schematically in Figure 1.

Surface Preparation and Protocol for PEEK-Ti with Hyaluronic Acid

Sixty mg of HA was sieved into 60 ml of double-distilled water to create the dipping solution, which was then mixed with 2.5 mg/ml 1-ethyl-3-(-3-dimethylaminopropyl) carbodiimide (EDC) and 0.63 mg/ml n-hydroxysuccinimide (NHS). After being submerged in this solution and reacting for six hours, the PEEK and nanoporous TiO_2_ PEEK samples were allowed to dry at room temperature [13]. The samples were named HA-coated PEEK and HA-coated nanoporous TiO_2_ PEEK following the HA coating.

Surface characterization of the samples

Attenuated Total Reflectance - Fourier Transform Infrared Spectroscopy (ATR-FTIR)

Nicolet 6700 FT-IR spectrometer using Omnic 8.0 software (ThermoFisher Scientific, Waltham, USA) recorded FT-IR spectra (600-4000 cm−1) of the sample (PEEK and HA-coated PEEK and HA-coated nanoporous TiO_2_ PEEK) in KBr pellets; 64 scans were accumulated with a spectral resolution of 2.0 cm−1. For the purpose of creating graphs, the spectra were exported in ASCII format to Origin 6.0 (Microcal Origin, Northampton, USA) software after being smoothed by 11 points and the baseline corrected. The second derivative algorithm was used to determine the shoulders' position.

Biological evaluation 

Cell Preparation

MG-63 cells (ATCC: C555) were purchased from Iran's National Center of Genetic Resources. To achieve the proper density, the cells were cultivated in Dulbecco's Modified Eagle Medium (DMEM) with 10% fetal bovine serum (FBS; GIBCO, New York, USA) and put in a cell incubator. Every three days, the cells' medium was changed.

To get rid of any remaining serum, the cells were first thoroughly rinsed with one milliliter of 1X phosphate-buffered saline (PBS) buffer after their prior media had been drained. Each flask was then filled with one milliliter of trypsin-ethylenediaminetetraacetic acid (EDTA; AS 9002-07-7, CAS 6381-92-6; Sigma, Nurnberg, Germany) solution, which contained 0.25% trypsin and 1 mM EDTA. The flasks were then incubated for three to five minutes or until the cells had separated from the culture vessel. After that, a 15 ml falcon tube was filled with the whole trypsin solution that contained the separated cells. Two milliliters of growth medium containing 10% FBS were added to suppress the trypsin function, and the mixture was centrifuged at 300 g for five minutes. Centrifugation produced a cell pellet, which was then suspended in 1 milliliter of culture media and cultivated in a T-75 flask.

Cell Viability Assay (MTT protocol)

The 3-[4,5-dimethylthiazol-2-yl]-2,5 diphenyl tetrazolium bromide (MTT) was added to PBS at a concentration of 5 mg/ml. The scaffolds were submerged in the culture media, and the cells were grown in triplicate, three-dimensional conditions, with roughly 5000 cells per well of a 96-well plate. The plate was incubated at 37°C for 72 hours and seven days to test the scaffolds' toxicity, and the supernatant was then taken out.

Each well received 100 microliters of MTT solution diluted to 1.10 (MTT starting stock: culture medium) in order to conduct this test. At 37°C, the plates were incubated for three to four hours. The formation of formazan causes the medium to turn purple. Following the removal of the cell supernatant, 100 microliters of dimethyl sulphoxide (DMSO)were applied to each well in order to dissolve the crystals that had formed. After that, they were kept in the incubator for fifteen minutes. After the DMSO solvent was solved, the scaffolds were taken out of the pipetted plate wells so that the plate could be read. A spectrophotometer was used to measure the absorbance at 570 nm.

The following groups were created, and they were cultured in an incubator set at 37°C for seven and twenty-one days: uncoated PEEK, HA-coated PEEK, HA and nanoporous TiO_2_ coated PEEK, and the control group, which contained osteoblast cells only.

DAPI Staining Protocol

Because of its stability and brightness, the DAPI (4′,6-diamidino-2-phenylindole) stain was chosen for identifying nucleoids; its excitation and emission maxima wavelengths are 358 and 461 nm. Both living and dead cells' nucleoids are stained with DAPI in blue (or white-blue). A whole-cell DAPI signal or the diffuse nature of DAPI staining in the nucleoid region indicates widespread harm to the cell population or genetic material.

As stated in the previous section, cells were cultivated for 21 days at 37°C and 5% CO_2 _with suitable medium changes after being seeded in 24-well plates on porous scaffolds for this test. After removing the media on day 21, the cells were fixed in a 4% paraformaldehyde solution (Merck, Darmstadt​​​​​​​, Germany) at 4°C for five minutes after being cleaned with 1X PBS. They were then rinsed with sterile distilled water, incubated for 10 minutes at 25 °C with 1 ug/mL DAPI stain (Millipore-Sigma, Burlington, USA), and then completely rinsed with sterile distilled water to get rid of any remaining dye. A fluorescent microscope (Olympus, Tokyo, Japan) with a magnification of ×400 (in the region of 133530 μm²) was used to visually check all samples using OLYSIA Bio Report Soft Imaging System GmbH, Version: 3.2 (Build 670).

Alkaline Phosphatase Activity 

Using a commercial enzyme-linked immunosorbent assay kit for human alkaline phosphatase (ALP; My BioSource, San Diego, USA), the rate at which the osteoblast cells produced ALP was determined in accordance with the manufacturer's instructions. In summary, 50,000 osteoblast cells were cultivated for 24 hours in a 24-well culture plate. The scaffold extraction solutions were added to the culture medium, which was then incubated for 21 days. The ALP kit reagent was then mixed with the homogenized osteoblast cells (containing 50 µg/μL of protein), and an enzyme-linked immunosorbent assay (ELISA) reader (Biotek Plate Reader, Winooski, USA) was used to measure the absorbance at 450 nm. 

Alizarin Red Staining

The calcium deposits released by MG-63 cells were measured using the Alizarin red S staining method. Following seven and 21 days of incubation, the cells were rinsed with PBS, fixed in 4% paraformaldehyde (Merck, Darmstadt​​​​​​​, Germany) for 10 minutes at 4 °C, and then stained for 10 minutes with 0.5% Alizarin red S (Sigma-Aldrich, Burlington, USA) in PBS. Two measurements were taken into consideration. They were then rinsed with sterile distilled water and incubated for 30 minutes at 25°C in 1 ml of 2% AlizarinrRed S solution (pH: 4.1-4.3; Millipore-Sigma, Burlington, USA). The bounded Alizarin red was then dissolved with 10% cetylpyridinium chloride (CPC) in 10 mM Na2HPO4 (pH=7) for two hours at 37°C. Using Gen5 (v:2.09.2) software, optical absorption was measured at 550 nm in a spectrophotometer equipped with a microplate reader (Biotek Plate Reader, Winooski, USA) [9].

Cell Attachment (Scanning Electron Microscopy) Study

As stated in the previous section, cells were cultivated for 21 days at 37 °C and 5% CO_2_ with suitable medium changes after being seeded in 24-well plates on porous scaffolds for this test. After removing the media on day 21, the cells were fixed with a freshly made 2.5% glutaraldehyde solution (Sigma-Aldrich, Burlington, USA) at 4˚C for 24 hours after being cleaned with 1X PBS. The samples were fixed in 1% osmium tetroxide (Sigma-Aldrich, Burlington, USA) at 25˚C for two hours after being submerged in PBS for the entire night. Ethanol at increasing concentrations of 30, 70, 80, 90, and 100% was used for dehydration. The samples were mounted on aluminum foil and then covered with a layer of gold. After that, a scanning electron microscope​​​​​​​ (SEM; VEGA\TESCAN, Brno​​​​​​​, Czech Republic) was used to examine the sample structures.

Statistical analysis

Version 26 of IBM's Statistical Package for the Social Sciences (SPSS; Armonk, US) program was utilized. The parametric tests were performed using the Kolmogorov-Smirnov (K-S) test. To determine statistical significance, one-way ANOVA followed by least significant difference post hoc testing was utilized. The threshold for statistical significance was p<0.05.

## Results

ATR-FTIR studies

**Figure 2:**
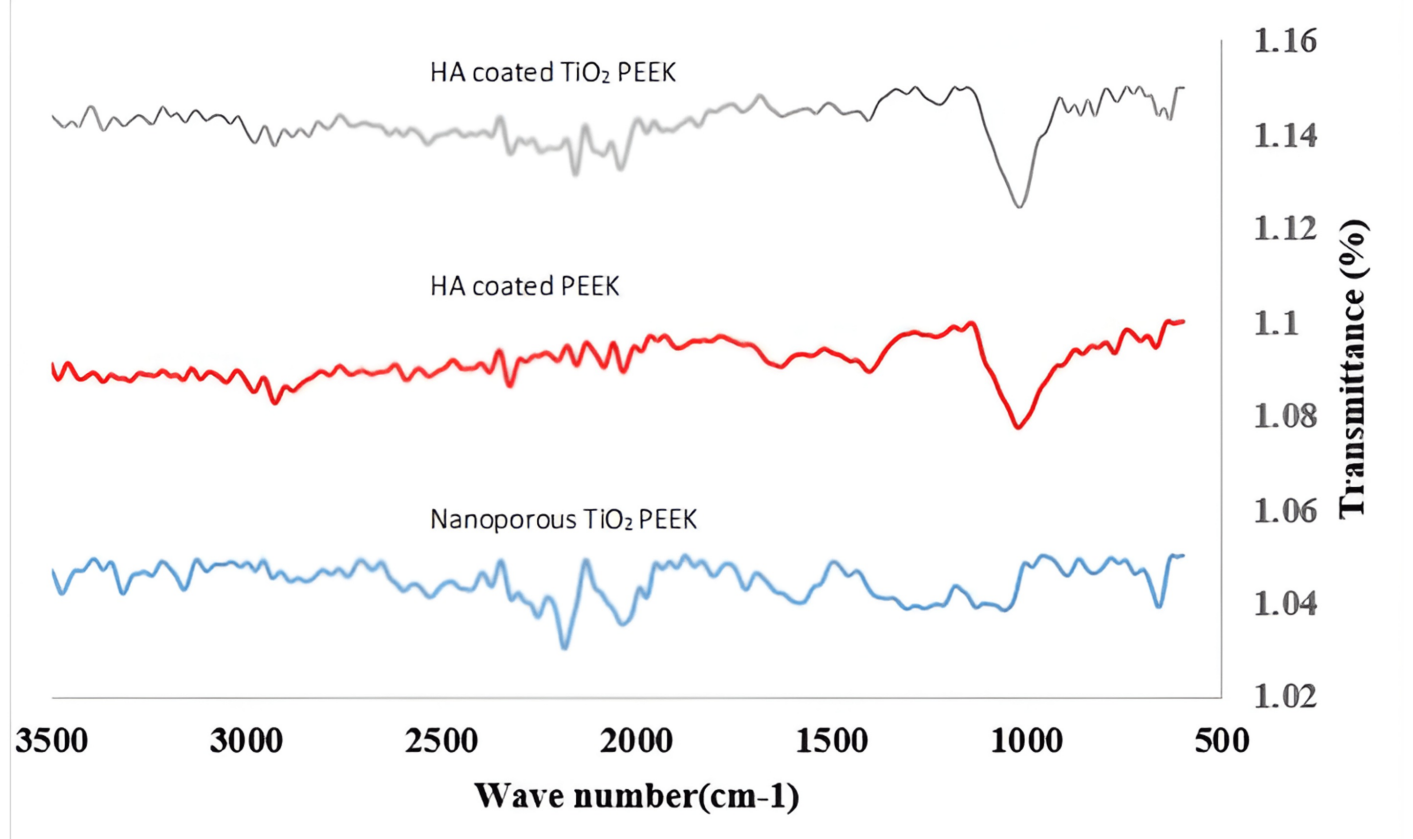
FTIR spectrum of different studied groups Transmittance drops (blue line) at 1049.12 cm⁻¹ as a result of increased absorption. TiO_2_ is characterized by Ti-O-Ti stretching vibrations. The PEEK-HA interaction is shown using HA-coated PEEK (red line). Retained peaks in the 1570 cm⁻¹ (C=O stretching) and 2985 cm⁻¹ (C-H stretching) ranges demonstrate the structural integrity of PEEK polymer. Across all wavenumbers, the transmittance of the gray line is lower than that of the components. FTIR - fourier transform infrared spectroscopy; PEEK - polyetheretherketone; Ti - titanium; HA - hyaluronic acid; TiO_2_ - titanium oxide; C=O - carbon=oxygen; C-H - carbon-hydrogen

Figure 2 shows the FTIR spectrum of nanoporous TiO_2_ PEEK, HA-coated PEEK, and HA-coated nanoporous TiO_2_ PEEK characterized by distinct peaks, each corresponding to the inherent functional groups of the constituent materials. There is a significant dip (blue line) in transmittance (higher absorption) in the region around 1049.12 cm⁻¹. This corresponds to the Ti-O-Ti stretching vibrations, which are characteristic of TiO_2_. Regarding the HA-coated PEEK, the red line shows the interaction between PEEK and HA. The PEEK polymer remains structurally intact, as indicated by the preserved peaks in the 1570 cm⁻¹ range (carbon=oxygen (C=O) stretching) and the 2985 cm⁻¹ range (carbon-hydrogen (C-H) stretching). The gray line shows generally lower transmittance across the entire wavenumber range compared to the individual components.

Biological evaluations

Table 1 shows the biological evaluation of the different study groups.

**Table 1:** Biological test results of the different studied groups PEEK - polyetheretherketone; HA - hyaluronic acid; TiO_2_ - titanium oxide; MTT - 3-[4,5-dimethylthiazol-2-yl]-2,5 diphenyl tetrazolium bromide; DAPI - 4',6-diamidino-2-phenylindole; ALP - alkaline phosphatase

Test	Control group	PEEK	HA-coated PEEK	HA-coated nonporous TiO_2_ PEEK
MTT assay				
Level 72 hours				
Mean ± SD	1.00 ± 0.04	0.95 ± 0.05	1.01 ± 0.02	0.92 ± 0.04
Median (Min - Max)	1.01 (0.92 - 1.06)	0.95 (0.88 - 1.04)	1.02 (0.98 - 1.06)	0.93 (0.87 - 1.02)
Level 7 days				
Mean ± SD	1.00 ± 0.01	0.99 ± 0.07	1.14 ± 0.07	1.10 ± 0.40
Median (Min - Max)	0.99 (0.98 - 1.02)	1.02 (0.88 - 1.02)	1.15 (1.02 - 1.24)	1.20 (0.13 - 1.39)
DAPI staining assay				
Level 21 days				
Mean ± SD	36.37 ± 4.56	53.25 ± 2.37	60.12 ± 2.41	69.37 ± 6.16
Median (Min - Max)	35.50 (32.0 - 46.0)	53.50 (49.00 - 57.00)	60.50 (55.00 - 63.00)	71.50 (55.0 - 74.0)
ALP				
Level 7 days				
Mean ± SD	0.17 ± 0.08	0.45 ±0.11	0.62 ± 0.17	0.59 ± 0.15
Median (Min - Max)	0.19 (0.07 - 0.30)	0.46 (0.30 - 0.65)	0.63 (0.38 - 0.88)	0.54 (0.45 - 0.87)
Level 21 days				
Mean ± SD	0.32 ± 0.16	0.84 ± 0.24	1.43 ± 0.16	2.18 ± 0.23
Median (Min - Max)	0.29 (0.19 - 0.70)	0.86 (0.41 - 1.12)	1.42 (1.24 - 1.71)	2.21 (1.80 - 2.50)
Alizarin red staining				
Level 7 days				
Mean ± SD	1.00 ± 0.09	1.20 ± 0.10	1.24 ± 0.11	1.41 ± 0.07
Median (Min - Max)	1.00 (0.90 - 1.14)	1.19 (1.05 - 1.39)	1.23 (1.11 - 1.46)	1.42 (1.31 - 1.55)
Level 21 days				
Mean ± SD	1.00 ± 0.05	1.01 ± 0.08	1.35 ± 0.07	1.72 ± 0.11
Median (Min - Max)	1.01 (0.91 - 1.09)	1.03 (0.86 - 1.13)	1.36 (1.23 - 1.45)	1.71 (1.54 - 1.90)

Cell Viability, Cell Proliferation and Cell Attachments

**Figure 3:**
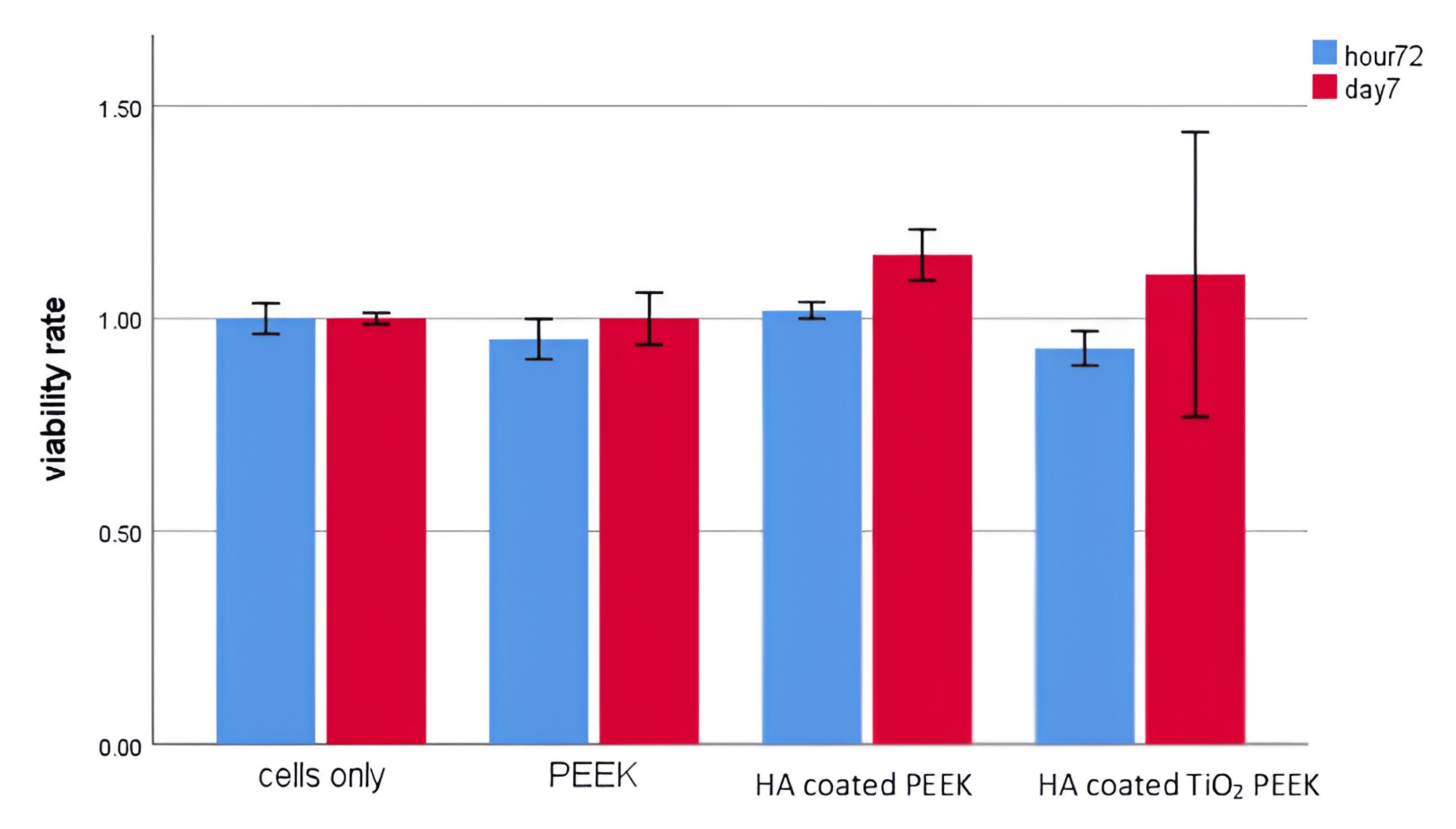
The vitality rates of MG-63 cell lines cultivated on study groups at two distinct time points: 72 hours (blue) and seven days (red) PEEK - polyetheretherketone; HA - hyaluronic acid; TiO_2_ - titanium oxide

Figure 3 shows cell viability rate at 72 hours (blue bars) and seven days (red bars) across different groups. The cells-only group maintains consistent viability at both time points, serving as a control. The PEEK group shows no significant change in viability, indicating that PEEK alone does not affect cell growth negatively or positively. In the HA-coated PEEK group, cell viability slightly increases, especially on the seventh day, suggesting that the HA coating improves cell compatibility over time. The most significant increase is observed in the HA-coated nanoporous TiO_2_ PEEK group, where the viability on the seventh was higher, albeit with larger variability, indicating that the combination of TiO₂ and HA promotes better cell survival and growth over time, possibly due to enhanced bioactivity and biocompatibility of the surface.

**Figure 4:**
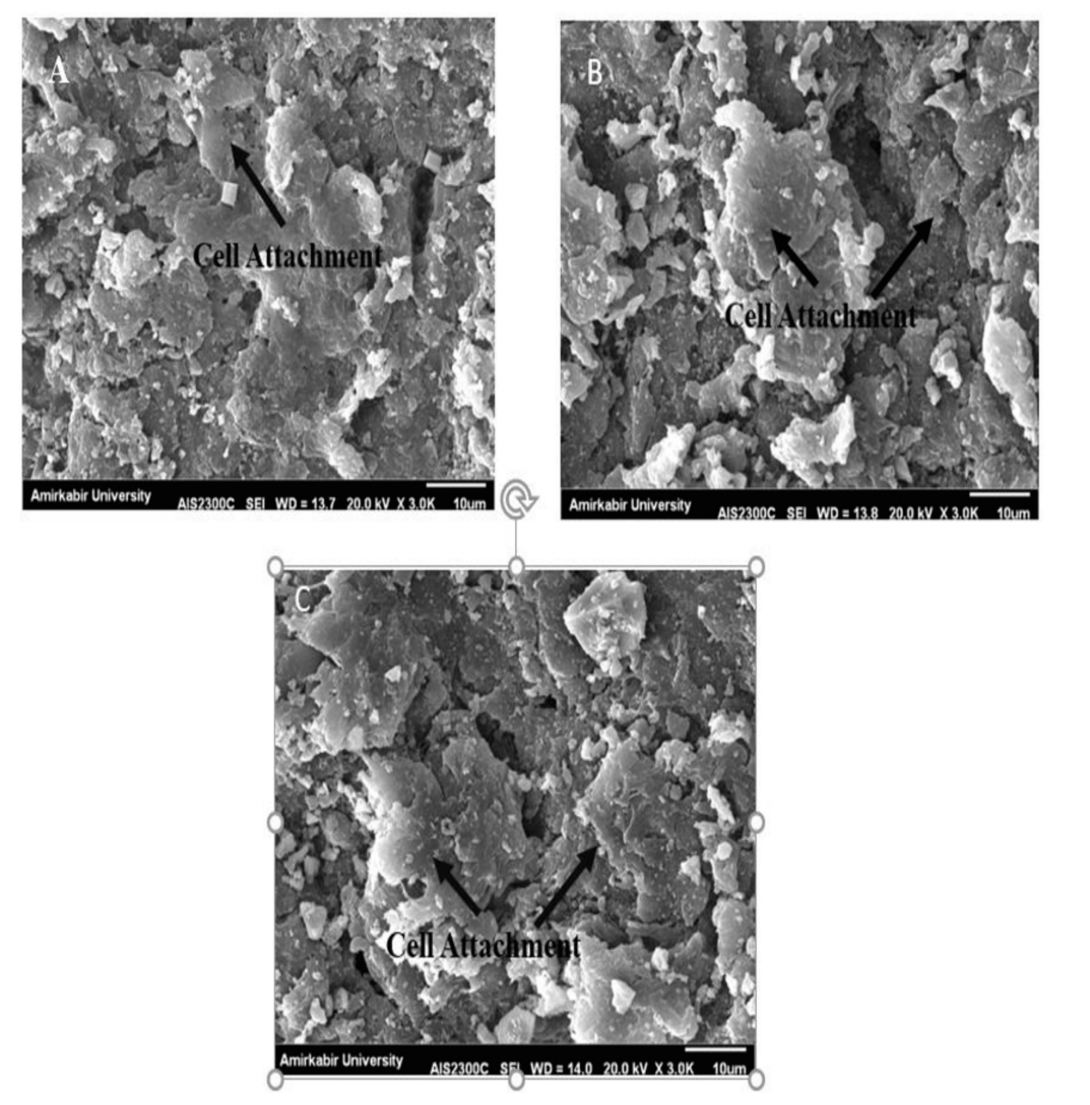
Scanning electron microscope showing cell attachments The cells attached to the scaffold were in the form of bone cells with short appendages and large cytoplasm, and around the cells, calcium deposits with a higher density than the cells were visible. A: In the PEEK group, the population of these cells was lower than compared to other groups. This amount was increased in the groups containing HA. B: In the HA-coated group, this population showed an increase compared to the uncoated group. C: This increase was the highest in the HA-coated nanoporous TiO_2 _PEEK group. PEEK - polyetheretherketone; HA - hyaluronic acid; TiO_2_ - titanium oxide

HA surface treatment with nanoporous TiO_2 _enhanced MG-63 cell numbers and biocompatibility, which was statistically significant compared to the control group (p=0.001), PEEK (p=0.001), and HA-coated PEEK (p=0.001). The growth of HA-coated PEEK cells was substantially greater than control cells (p=0.001) and PEEK cells (p=0.01). Phalloidin/DAPI immunofluorescence labeling showed the proliferation of cells on HA-coated and untreated PEEK surfaces (Figure 4).

ALP Activity

**Figure 5:**
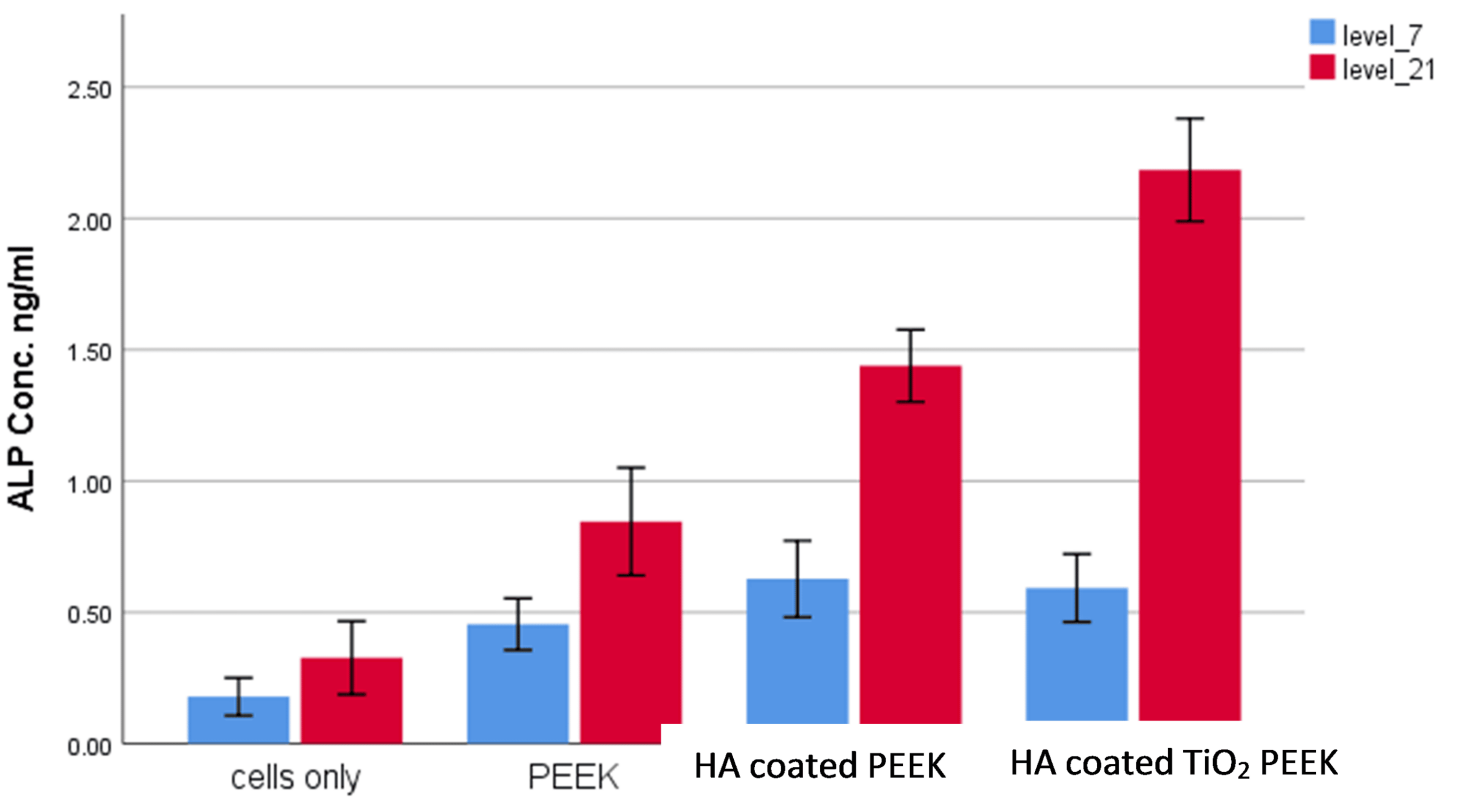
ALP activity for the MG-63 cell line cultivated in different study groups The absorbance at 450 nm is the measure of ALP activity. Each sample's ALP level was tested on days 7 (blue) and 21 (red). The ALP activity of the HA-coated nanoporous TiO_2_ PEEK group was greater than that of the other three groups (p˂0.001) on day 21 (red line) followed by the HA-coated PEEK (p˂0.001). PEEK - polyetheretherketone; HA - hyaluronic acid; TiO_2_ - titanium oxide; ALP - alkaline phosphatase

Figure 5 shows the ALP findings for the study groups at two time points: seven days (blue bars) and 21 days (red bars). ALP activity is an important measure of osteogenic differentiation, demonstrating how effectively the cells are moving toward bone formation. The cell-only group had the lowest ALP activity at both levels, suggesting limited osteogenic activity. The PEEK group had a slight rise in ALP activity, showing that PEEK offers a favorable environment for cell proliferation but does not considerably improve osteogenic differentiation. ALP activity rises further in the HA-coated PEEK group, particularly at the level of 21 days, indicating that the HA coating promotes osteogenic differentiation by increasing cell contact and adhesion. The HA-coated nonporous TiO_2_ PEEK group has the greatest ALP activity, notably at the level of 21 days, indicating a robust osteogenic response.

Alizarin Red Staining

**Figure 6:**
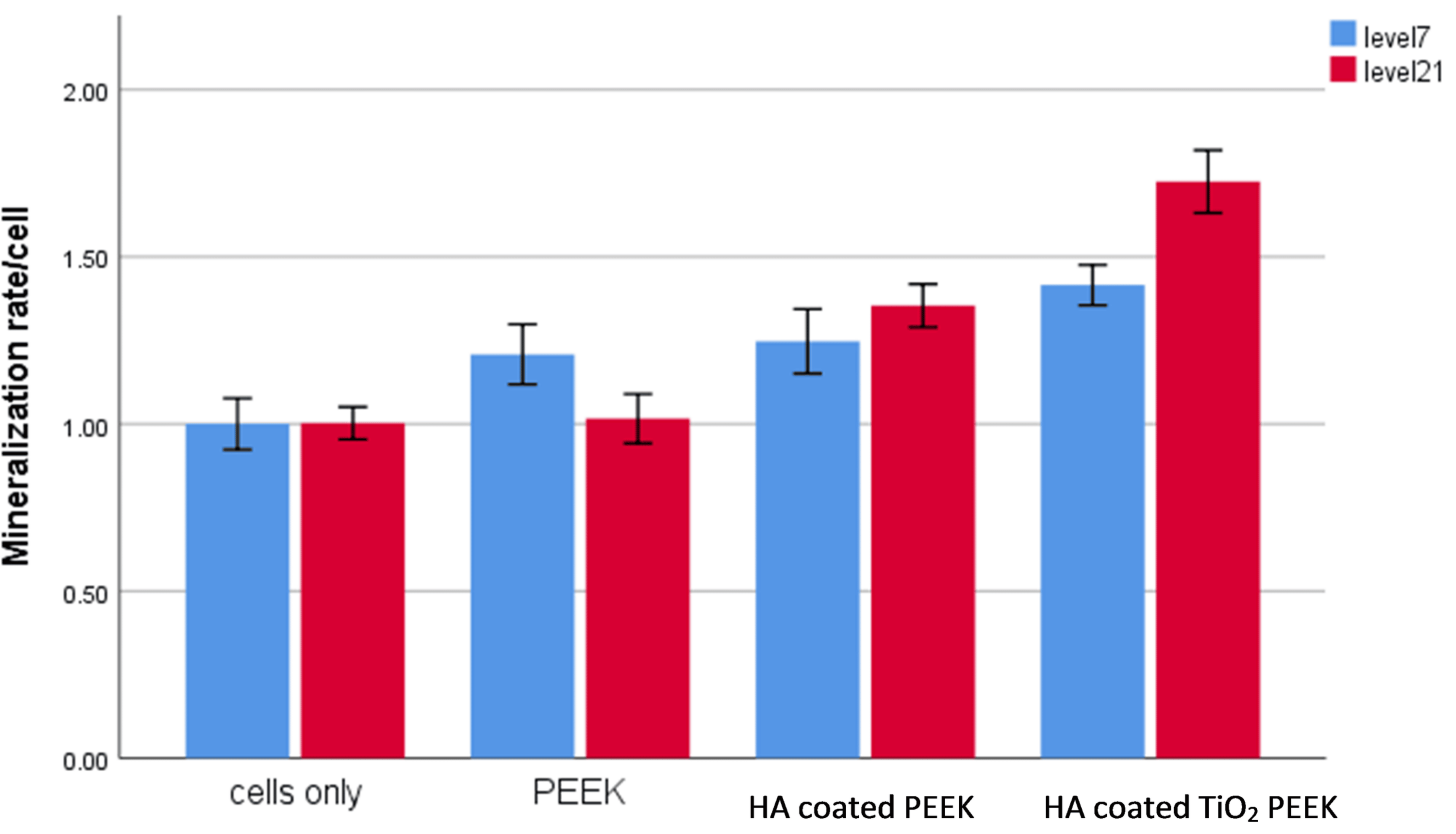
Colorimetric quantification of the extracellular matrix mineralization at seven days (blue) and 21 days (red) HA-coated nanoporous TiO_2_ PEEK has a higher level than control, PEEK and HA-coated PEEK groups (p<0.05). PEEK - polyetheretherketone; HA - hyaluronic acid; TiO_2_ - titanium oxide; ALP - alkaline phosphatase

Figure 6 shows Alizarin staining results, which measure calcium deposition - a key marker for bone mineralization and osteogenesis - at two time points: level seven (blue bars) and level 21 (red bars). The cells-only group shows minimal calcium deposition at both levels, serving as the baseline. The PEEK group shows slightly higher levels of calcium deposition compared to the control in a manner that was significant at the level of seven days (p˂0.001), while this level was decreased to a point that was not significant after 21 days (p=0.9). The HA-coated PEEK group shows a further increase in calcium deposition, particularly at level 21 (p˂0.001). The highest calcium deposition was observed in the HA-coated nonporous TiO_2_ PEEK group, especially at the level of 21 days (p˂0.001) compared to other groups.

## Discussion

It is widely known that the surface properties of biomaterials affect the growth capacity of MG-63 cells, a human osteosarcoma cell line. The properties of the biomaterial surfaces, such as their composition, shape, roughness, hydrophilicity, and functional groups, significantly affect the activities and capacities of osteoblasts and the bone-formation process [14,15].

PEEK, although biocompatible, is often considered physiologically inert, indicating that it does not substantially improve cellular interactions or survivability when used alone. Nevertheless, some surface alterations may enhance these characteristics [16,17].

In this research, we successfully coated the PEEK implant surface with nanoporous TiO_2_ and HA. In a previous investigation, for the first time in the literature, we coated HA on PEEK surface with a promising result [18]. However, in order to develop the biocompatibility and hydrophilicity of the PEEK material surface further, we added nanoporous TiO_2_ beside the HA. The addition of nanoporous TiO_2_ to PEEK has been documented in the literature with positive results [19]. HA may enhance hydrophilicity and facilitate cell adhesion, thereby improving cell survival and proliferation, particularly in tissue engineering applications [20]. In a comparable way, the incorporation of TiO₂ results in the formation of a bioactive surface that further enhances cell attachment and growth. TiO₂, when incorporated into PEEK, enhances surface roughness and wettability, thereby promoting cell adhesion and subsequent rapid growth [19]. Consequently, the maximum levels of both cell viability and osteogenic differentiation can be observed when TiO₂ and HA are combined with PEEK.

In the present study, in all of the biological tests, the cell viability, proliferation, differentiation, and mineralization of osteoblasts in the HA-coated group was higher than in the PEEK and cells-only groups. Moreover, the results of mixed coating with nanoporous TiO_2_ and HA were significantly higher than the HA-coated group and uncoated PEEK group. However, the viability test could be seen as an exception since the mixed-coated group's viability was slightly lower than the HA-coated group only in the period of 72 hours, although the difference was not statistically significant. This could be attributed to the size of the particles, the non-uniform coating of the HA, and a higher degree of bacterial colonization [21].

Our ALP and Alizarin results demonstrated that the HA-coated nanoporous TiO_2_ PEEK substrate significantly promotes osteogenesis compared to both uncoated PEEK and HA-coated PEEK, consistent with the findings reported in other studies [22,23].

The findings of this investigation regarding the HA-coated nanoporous TiO_2_ PEEK exhibited superior mineralization and osteoblast differentiation. This may be ascribed to the combined effects of HA and the nanogranular structured TiO_2_, which helped to modify the surface energy and hydrophilicity. Certain HA-coated biomaterials may be biocompatible with a variety of cells, according to literature reports [24,25].

HA, known for its biocompatibility, mechanical properties, and gradual degradation, is widely used in medical fields like tissue engineering, promoting bone growth, cell adhesion, and angiogenesis [11,25].

Aggrecan, neurocan, and versican are among the molecules with which HA interacts, besides a number of receptors, including the cell surface glycoprotein CD44, the hyaluronan-mediated motility receptor (RHAMM), toll-like receptors (TLRs), glial hyaluronate-binding protein (GHAP), intracellular adhesion molecule-1 (ICAM-1), and lymphatic vessel endothelial HA receptor (LYVE-1) [26]. The receptors in mesenchymal cells that have drawn the greatest interest are CD44, RHAMM, and TLR4 (toll-like receptor 4). CD44, a flexible glycoprotein found in cell membranes, is the primary HA receptor. Numerous biological processes, such as cell division, proliferation, and bone metabolism, are impacted by the interaction between HA and CD44 [27]. As a result, the proper genes are activated, and cell differentiation and proliferation are started [18].

Finally, the role of TiO₂ is crucial in enhancing osseointegration, the biological bonding process between bone and implants. It is commonly used to modify titanium implant surfaces, creating a nano-roughened topography that significantly boosts cell attachment and osteoblast proliferation. This increased surface area and roughness facilitate better adherence and growth of bone cells, thereby accelerating osseointegration [28]. Research indicates that TiO₂ coatings improve both the mechanical stability and bioactivity of implants, with TiO₂ nanotubes particularly effective in promoting the differentiation of mesenchymal stem cells into osteoblasts [29,30].

The strength of this study was the successful coating of nanoporous TiO_2_ and HA on the PEEK surface, which was supported by numerous biological tests. However, it has some limitations also. The study used an osteoblast-like cell line to study the complex biological traits of real osteoblasts. Further research is needed to determine the best hyaluronic acid content for coatings, optimize effectiveness, and compare high and low molecular weight hyaluronic acid for cell survival, proliferation, differentiation, and mineralization.

## Conclusions

This study reveals that the addition of HA and TiO₂ to PEEK improves cell viability, proliferations, attachments, differentiations, and mineralization, making it suitable for biomedical applications that require long-term cell integration. This synergistic effect makes the composite material promising for tissue integration applications like implants and tissue engineering scaffolds. Additionally, the combination of TiO₂ and HA enhances bone-forming activity.
